# Comparison of methods for early-readmission prediction in a high-dimensional heterogeneous covariates and time-to-event outcome framework

**DOI:** 10.1186/s12874-019-0673-4

**Published:** 2019-03-06

**Authors:** Simon Bussy, Raphaël Veil, Vincent Looten, Anita Burgun, Stéphane Gaïffas, Agathe Guilloux, Brigitte Ranque, Anne-Sophie Jannot

**Affiliations:** 1 Laboratoire de Probabilités Statistique et Modélisation (LPSM), UMR 8001, Sorbonne University, 4 Place Jussieu, Paris, 75005 France; 2 Assistance Publique-Hôpitaux de Paris, Biomedical Informatics and Public Health Department, European Georges Pompidou Hospital, 20 Rue Leblanc, Paris, 75015 France; 3 INSERM UMRS 1138, Eq22, Centre de Recherche des Cordeliers, Université Paris Descartes, 15 Rue de l’École de Médecine, Paris, 75006 France; 4 CMAP, UMR 7641 École Polytechnique CNRS, Route de Saclay, Palaiseau, 91128 France; 5 LAMME, Univ Evry, CNRS, Université Paris-Saclay, 23 boulevard de France, Evry, 91025 France; 6 INSERM UMRS 970, Université Paris Descartes, 56 rue Leblanc, Paris, 75015 France; 7 Assistance Publique-Hôpitaux de Paris, Internal Medicine Department, Georges Pompidou European Hospital, 20 Rue Leblanc, Paris, 75015 France

**Keywords:** Hospital readmission risk, High-dimensional prediction, Survival analysis, Machine learning methods, Sickle-cell disease

## Abstract

**Background:**

Choosing the most performing method in terms of outcome prediction or variables selection is a recurring problem in prognosis studies, leading to many publications on methods comparison. But some aspects have received little attention. First, most comparison studies treat prediction performance and variable selection aspects separately. Second, methods are either compared within a binary outcome setting (where we want to predict whether the readmission will occur within an arbitrarily chosen delay or not) or within a survival analysis setting (where the outcomes are directly the censored times), but not both. In this paper, we propose a comparison methodology to weight up those different settings both in terms of prediction and variables selection, while incorporating advanced machine learning strategies.

**Methods:**

Using a high-dimensional case study on a sickle-cell disease (SCD) cohort, we compare 8 statistical methods. In the binary outcome setting, we consider logistic regression (LR), support vector machine (SVM), random forest (RF), gradient boosting (GB) and neural network (NN); while on the survival analysis setting, we consider the Cox Proportional Hazards (PH), the CURE and the C-mix models. We also propose a method using Gaussian Processes to extract meaningfull structured covariates from longitudinal data.

**Results:**

Among all assessed statistical methods, the survival analysis ones obtain the best results. In particular the C-mix model yields the better performances in both the two considered settings (AUC =0.94 in the binary outcome setting), as well as interesting interpretation aspects. There is some consistency in selected covariates across methods within a setting, but not much across the two settings.

**Conclusions:**

It appears that learning withing the survival analysis setting first (so using all the temporal information), and then going back to a binary prediction using the survival estimates gives significantly better prediction performances than the ones obtained by models trained “directly” within the binary outcome setting.

**Electronic supplementary material:**

The online version of this article (10.1186/s12874-019-0673-4) contains supplementary material, which is available to authorized users.

## Background

Recently, many statistical developments have been performed to tackle prognostic studies analysis. Beyond accurate risk estimation, interpretation of the results in terms of covariates importance is required to assess risk factors, with the ultimate aim of developing better diagnostic and therapeutic strategies [37].

In most studies, covariate selection ability and model prediction performance are regarded separately. On the one hand, a considerable amount of studies report on covariates relevancy in multivariate models, mostly in the form of ajusted odds ratio [32] (for instance using logistic regression (LR) model [1, 34]) without reporting on the method’s prediction performance (goodness-of-fit and overfitting aspects are neglected); namely disregarding the question: *is the model prediction still accurate on new data, unseen during the training phase?* While on the other hand, most studies focusing on a method’s predictive performance do not mention its variable selection ability [21], thus making it not well suited for the high-dimensional setting. Such settings are becoming increasingly common in a context where the number of available covariates to consider as potential risk factors is tremendous, especially with the development of electronic health record (EHR).

In this paper, we discuss both aspects (prediction performance and covariates selection) for all considered methods, with a particular emphasis on the *Elastic-Net* regularization method [52]. Regularization has emerged as a dominant theme in machine learning and statistics. It provides an intuitive and principled tool for learning from high-dimensional data.

Then, a lot of studies consider prognosis as a binary outcome, namely whether the event-of-interest (death, relapse or hospital readmission for instance) occurs whithin a pre-specified period of time we denote *ε* [4, 41, 44, 47]. In the following, we refer to this framework as the *binary outcome setting*, and we denote *T*≥0 the time elapsed before the event-of-interest and $X \in \mathbb {R}^{d}$ the vector of *d* covariates recorded at the hospital during a stay. In this setting, we are interested in predicting *T*≤*ε*. Such an a priori choice for *ε* is questionable, since any conclusion regarding both prediction and covariates relevancy is completely conditioned on the threshold value *ε* [11]. Hence, it is hazardous to make general inference on the probability distribution of the time-to-event outcome given the covariates from such a restrictive binary prediction setting.

An alternative setting to model prognosis is the survival analysis one, that takes the quantitative censored times as outcomes. The time *T* is right censored since in practice, some patients have not been readmitted before the end of follow-up. In the following, we refer to this setting as the *survival analysis setting* [27] and we denote *Y* the right-censored duration, that is *Y*= min(*T*,*C*) with *C* the time when the patient is lost to follow-up. Few studies compare the survival analysis and binary outcome settings and none of them considers simultaneously the prediction and the variable selection aspects in a high dimensional setting. For instance in [11], only the Cox Proportional Hazards (PH) model [12] is considered in the survival analysis setting and a dimentionality reduction phase (or screening) is performed prior to the models comparison, as it is often the case [5, 13].

Our case study focuses on hospital readmission following vaso-occlusive crisis (VOC) for patients with sickle-cell disease (SCD). SCD is the most frequent monogenic disorder worldwide. It is responsible for repeated VOC, which are acute painful episodes, utlimately resulting in increased morbidity and mortality [9, 38]. Although there are some studies regarding risk factors of early complications, only a few of them specifically addressed the question of early-readmission prediction after a VOC episode [8, 40].

For a few decades, hospital readmissions have been known to be responsible for huge costs [18, 28]; they are also a measure of health care quality. Today, hospitals have limited ressources they can allocate to each patient. Therefore, identifying patients at high risk of readmissions is a paramount question and predictive models are often used to tackle it.

The purpose of this manuscript is to compare different statistical methods to analyse readmission, with the final goal to build decision tools for physician to help them identify patients at high risk of readmission. To make such comparisons, we consider both the predictive performance and the covariates selection aspect of each model, on the same high-dimensional set of covariates.

In the binary outcome setting, we consider LR [25] and support vector machine (SVM) [42] with linear kernel, being both penalized with the Elastic-Net regularization [52] to deal with the high dimensional setting and avoid overfitting [23]. We also consider random forest (RF) [7], gradient boosting (GB) [19] and artificial neural networks (NN) [50].

We then abstain from the a priori threshold choice and consider the survival analysis setting. We apply first the Cox PH model [12]. We also apply the CURE model [15, 30], that considers one fraction of the population as cured or not subject to any risk of readmimssion. Finally, we consider the recently developped high dimensional C-mix mixture model [10]. The three considered models in this setting are also penalized with the Elastic-Net regularization.

## Methods

### Motivating case study

We consider a monocentric retrospective cohort study of *n*=286 patients. George Pompidou University Hospital (GPUH) is an expertise center for SCD adult patients [31]. Data is extracted from the GPUH Clinical Data Warehouse (CDW) using the i2b2 star-shaped standard [51]. It contains routine care data divided into several categories among them demographics, vital signs, diagnoses (ICD-10 [49]), procedures (French CCAM classification [45]), EHR clinical data from structured questionnaires, free text reports, Logical Observation Identifiers Names and Codes (LOINC), biological test results, and Computerized Provider Order Entry (CPOE) drug prescriptions. The sample included all stays from patients admitted to the internal medicine department for VOC (ICD-10 57.0 or 57.2) between January 1st 2010 and December 31st 2015 and the follow-up was performed on the same period.

Over half of the patients has only one stay during the follow-up period (see Section 2.1 of Additional file 1). We hence randomly sample one stay per patient and focus on the early-readmission risk afterwards. This enables us, in addition, to work on the *independent and identically distributed* standard statistical framework.

### Covariates

We extracted demographic data (e.g. sex, date of birth, last known vital status), as well as both qualitative (e.g. the admission at any point during the stay to an ICU, the type of opioid drug received) and quantitative time-dependent variables (e.g. biological results, vital sign values, intraveinous opiod syringes parameters) regarding each stay.

We also extracted all the free text reports from the patients’ EHR regardless of the source department and the stay. In order to facilitate variable extraction from such textual reports, we used a locally developed browser-accessible tool called FASTVISU [14]. This software is linked with the CDW, and allowed us to quickly check throughout these textual reports for highlighted information and to vote for variable status (e.g. SCD genotype) or value (e.g. baseline hemoglobinemia). Keywords using regular expressions are used to focus on specific mentions within the text. Variables extracted using this tool were the following: SCD genotype, baseline hemoglobinemia, medical history (with a focus on previous VOC complications and SCD-related chronic organ damages), and lifestyle related information. For time-dependent variables, status was determined per stay, including the ones that were not related to a VOC episode (e.g. annual check-ups).

We extracted for the included patients all stays encoded as VOC to derive time length from and until the respectively previous and consecutive stays. Regarding demographic data, we derived the patient’s age at admission for each stay. For each time-dependent covariate, all patient relative time series have different number of points and different length. We then propose a method to extract several covariates from each time series, to make the use of usual machine learning algorithms possible:

- Regarding all vital parameters and oxygen use, we derived them by calculating the average value and the linear regression’s slope for the last 48 h of the stay, as well as the delay between the end of oxygen support and the hospital discharge.
- Regarding biological variables, we only kept the ones that were measured at least once for more than 50% of the stays. We considered the last measured value for each of them before discharge. Additionally, for covariates with at least 2 distinct measurements per stay, we considered the linear regression’s slope for the last 48 h of the stay. In order to maximize the amount of biological data, we also retrieved the biological values measured in the emergency department, prior to the administrative admission of the patient.
- For each time-dependent covariate and for each stay, we fit a distinct Gaussian process on the last 48 h of the stay for all patient with at least 3 distinct measurements during this period, and extract the corresponding hyper-parameters as covariates for our problem.

Indeed, Gaussian processes are known to fit EHR data well; see for instance [36], where a distinct Gaussian process is also fitted for each patient and each time-dependent covariate, in order to cluster patients into groups in the hyper-parameter space. In our study, we instead use the hyper-parameters as covariates in a supervised learning way. We use Gaussian process with linear average function and a sum-kernel composed by a constant kernel which modifies the mean of the Gaussian process, a radial-basis function kernel, and a white kernel to explain the noise-component of the signal.

After a binary encoding of the categorical covariates, the final dimension of the working space (number of considered covariates) is *d*=174. Therefore, the number of patients is less than 2 times as many as the number of covariates, making it difficult to use standard regression techniques. More details on data extraction, missing data imputation, as well as a precise list of all considered covariates, are given in Sections 2.2, 2.3 and 2.4 (given in Additional file 1) respectively.

### Statistical methods and analytical strategies

#### Binary outcome setting

**Fig. 1:**
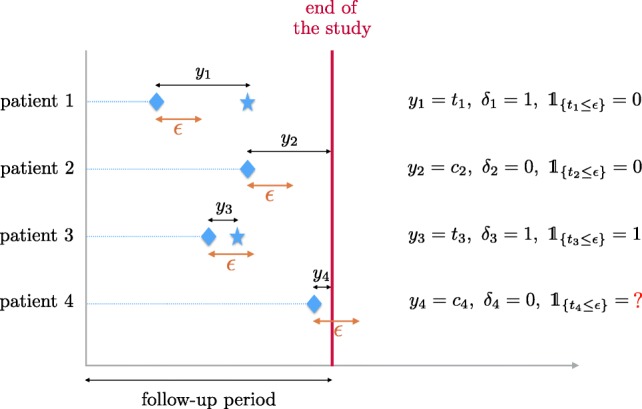
Illustration of different situations when dealing with censored data that cannot be labeled when using a threshold *ε*. $\delta _{i} = \mathbbm{1}_{\{T_{i} \leq C_{i}\}}$ is the censoring indicator which is equal to 1 if *Y*_*i*_ is censored and 0 otherwise. In the binary outcome setting, patient 4 would be excluded

In this setting, we consider as early-readmission any readmission occuring within 30 days of hospital discharge after a previous hospital stay for VOC, the 30 days threshold being a standard choice in SCD studies [8, 17]. A first drawback of this setting (which is rarely mentionned) is that patients having both a censored time and *c*_*i*_≤*ε* have to be excluded from the procedure, since we do not know if *t*_*i*_≤*ε* or not. Figure 1 gives an illustration of this last point. In our case, 7 patients have to be excluded because of this issue.

Seven patients had a follow-up period below 30 days while they were not readmitted during this period. Therefore, in the binary outcome setting, it was not possible to label them as readmitted or not since they could have been readmitted after the end of the study but within their first 30 days after hospital discharge. Consequently, we had to excluded them in this setting.

For retrospective studies with short *ε* delay, it is often possible to label those patients looking at what happened after the end of the study. But strictly in terms of methodology, we act in this paper as if we do not have any information after the end of the study (which can be viewed as the future), following the survival analysis framework [16]. This technical problem always occurs when considering a threshold delay to obtain binary outcomes from censored times, but we did not found any paper mentioning it.

We first consider LR [25] and linear kernel SVM [42], both penalized with the Elastic-Net regularization [52]. For a given model, using this penalization means adding the following term $\gamma \left ((1-\eta)\| \beta \|_{1} + (\eta /2) \| \beta \|_{2}^{2} \right)$ to the cost function (the negative likelihood for instance) in order to minimize it in $\beta \in \mathbb {R}^{d}$, a vector of coefficients that quantifies the impact of each biomedical covariates on the associated prediction task. This means that the Elastic-Net regularization term is a linear combination of the lasso (*ℓ*_1_) and ridge (squared *ℓ*_2_) penalties for a fixed *η*∈(0,1), tuning parameter *γ*, and where we denote $\| \beta \|_{p} = \left (\sum _{i=1}^{d} |\beta _{i}|^{p} \right)^{1 / p}$ the *ℓ*_*p*_-norm of *β*. One advantage of this regularization method is its ability to perform model selection (for the lasso part) and to pinpoint the most important covariates relatively to the prediction objective. On the other hand, the ridge part allows to handle potential correlation between covariates [52]. The penalization parameter *γ* is carefully chosen using the same cross-validation procedure [29] for all competing models. Note that in practice, the intercept is not regularized.

We also consider other machine learning algorithms in the ensemble methods class such as RF [7] and GB [19]. For both algorithms, all hyper-parameters are tuned using a randomized search cross-validation procedure [2]. For instance for RF: the number of trees in the forest, the maximum depth of the tree or the minimum number of samples required to split an internal node. Note also that regarding the covariates importance for RF and GB, we use the Gini importance [33], defined as the total decrease in node impurity weighted by the probability of reaching that node (which is approximated by the proportion of samples reaching that node) averaged over all trees of the ensemble. That is why the corresponding coefficients are all positive for those two models, which is to be kept in mind. Finally, we consider NN [50] in the form of a multilayer perceptron neural network with one hidden layer. We use stochastic gradient-based optimizer for NN and rectified linear units activation function to get sparse activation and be able to compare covariate importance [20]. The regularization term as well as the number of neurons in the hidden layer are also cross-validated though a random search optimization. Note that many studies in the literature choose hyper-parameters of the models, without mentioning any statistical procedure to determine them without a priori [39].

For all considered models in this setting, we use the reference implementations from the scikit-learn library [35].

#### Survival analysis setting

The Cox PH model is by far the most widely used in the survival analysis setting; see [12] and [43] for the penalized version. It is a regression model that describes the relation between intensity of events and covariates, given by *λ*(*t*)=*λ*_0_(*t*)exp(*x*^⊤^*β*) where *λ*_0_ is a baseline intensity describing how the event hazard changes over time at baseline levels of covariates, and *β* is a vector quantifying the multiplicative impact on the hazard ratio of each covariate. We use the R packages survival and glmnet to train this model. An alternative to the Cox PH model is the CURE model [15] with an Elastic-Net regularization, that considers one fraction of the population as not subject to any risk of readmission, with a logistic function for the incidence part and a parametric survival model. Finally, we apply the C-mix model [10] that is designed to learn risk groups in a high dimensional survival analysis setting. For a given patient *i*, it provides a marker $\pi _{\hat {\beta }}(x_{i})$ estimating the probability that the patient is at high risk of early-readmission. Note that $\hat {\beta }$ denotes the estimate vector after the training phase for any model. The C-mix (as well as CURE as a particular case) high-dimensional implementation is available online as an open-source project at https://github.com/SimonBussy/C-mix. We point this out since all other methods used in this manuscript are readily accessible in almost all development framework, which is not the case for the C-mix model.

We randomly split data into a training set and a test set (30% for testing, cross-validation is done on the training). In both binary outcome and survival analysis settings, all the prediction performances are evaluated on the test set after the training phase, using the relevant metrics detailed hereafter. Note also that for all considered models (except RF and GB), continuous covariates are standardized through a preprocessing step, which allows proper comparability between the covariates’ effects whithin each model.

### Metrics used for analysis

In the binary outcome setting, the natural metric used to evaluate performances is the AUC [6]. In the survival analysis setting, the natural equivalent is the C-index (implemented in the python package lifelines), that is $\mathbb {P}\left [M_{i} > M_{j} | Y_{i} < Y_{j}, Y_{i} < \tau \right ]$ with *i*≠*j* two independent patients, *τ* corresponding to the follow-up period duration [24], and *M*_*i*_ the natural risk marker of the model for patient *i*: $\exp \left (x_{i}^{\top } \hat {\beta }\right)$ for the Cox PH model, the probability of being uncured for the CURE model and $\pi _{\hat {\beta }}(x_{i})$ for the C-mix.

To compare the two settings, we use the estimated survival function $\hat {S}_{i}$ for each model and patient *i* in the test set. Then, for a given threshold *ε*, we now use the estimated probability $\hat {S}_{i} \left (\epsilon |X_{i} = x_{i} \right) \in [0, 1]$ for each model to predict whether or not *T*_*i*_≤*ε*∈{0,1} on the test set – relaying to the binary outcome setting – thus assessing performances using the classical AUC score. Then, with *ε*=30 days, one can directly compare prediction performances with those obtained in the binary outcome setting. We refer to this technique as $\hat {S}^{\text {model}}$ in Table 1, with “model” the appropriate survival analysis model. Details on the survival function estimation for each model are given in Section 3.1 of Additional file 1.

**Table 1:** Comparison of prediction performances in the two considered settings, with best results in bold

Setting	Metric	Model	Score
Survival analysis	C-index	CURE	0.718
		Cox PH	0.725
		C-mix	**0.754**
Binary outcome	AUC	SVM	0.524
		GB	0.561
		LR	0.616
		NN	0.707
		RF	0.738
		$\hat {S}^{\text {CURE}}$ (*ε*=30)	0.831
		$\hat {S}^{\text {Cox}}$ (*ε*=30)	0.855
		$\hat {S}^{\text {C-mix}}$ (*ε*=30)	**0.940**

Finally, we compute the pairwise Pearson correlation between the absolute (because of the positive vectors for RF and GB) covariates importance vectors of each method to obtain a similarity measure in terms of covariates selection [26].

## Results

Table 1 compares the prediction performances of the different methods in both considered settings using appropriate metrics. For the binary outcome setting, results in terms of accuracy and F-measure are also given in Section 4 of Additional file 1. Corresponding hyper-parameters obtained by cross-validation are detailed in Section 3.2 of Additional file 1.

**Fig. 2:**
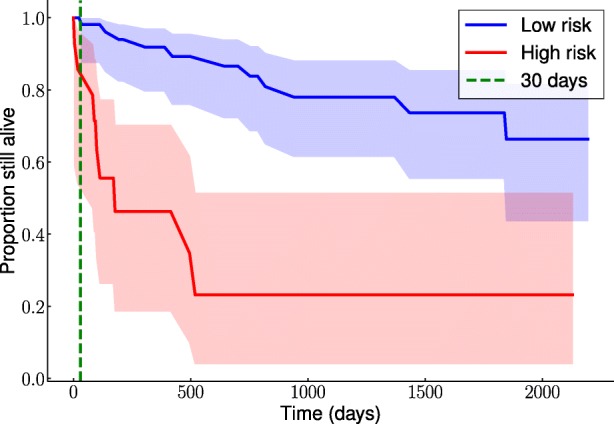
Estimated survival curves per subgroups (blue for low risk and red for high risk) with the corresponding 95% confidence bands

Thus, making binary predictions from survival analysis models using estimated survival function highly improves performances. Among all considered survival analysis models, the C-mix yields the best results. Figure 2 displays the estimated survival curves for the low and high risk of early-readmission subgroups learned by this model. Note the clear separation between the two subgroups.

**Fig. 3:**
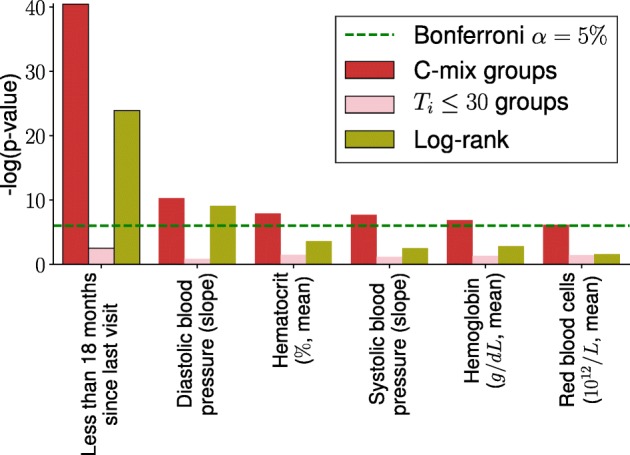
Comparison of the tests based on the C-mix groups, on the *ε*=30 days relative groups and on survival times. We arbitrarily shows only the tests with corresponding *p*-values below the level *α*=5*%*, with the classical Bonferroni multitests correction [3]

Based on those early-readmission risk learned subgroups, we test for significant differences between them using Fisher-exact test [46] for binary covariate, and Wilcoxon rank-sum test [48] for quantitative covariate. Then, we similarly test for significant difference, on each covariate, between naively created groups obtained with the binary outcome setting (*ε*=30 days). We also use the log-rank test [22] on each covariate, directly involving quantitative readmission delays. Finally, we compared the obtained significance (the *p*-value) for each test, on each covariate. The tests induced by the C-mix model are the most significant ones for almost all covariates. The top-6 *p*-values of the tests are compared in Fig. 3.

Taking the most significant C-mix groups highlighted in Figs. 3 and 6 shows either boxplot (for quantitative covariates) or repartition (for qualitative covariates) comparison between those groups. One can now easily visualize and interpret early-readmission risk data-driven grouping, and focus on specific covariate. For instance, it appears that patients among the high risk group tend to have a lower hemoglobin level, as well as a slightly lowering diastolic blood pressure in the last 48 h of the stay (while slightly uppering for the low risk group). It also appears that less patients among the low risk group have visited the emergency department in the last 18 months.

**Fig. 4:**
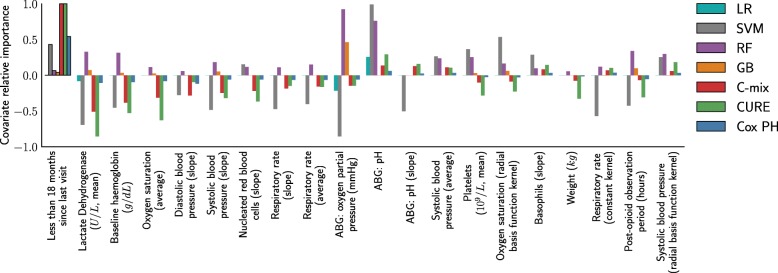
Comparison of the top-20 covariates importance ordered on the C-mix estimates. Note that some time-dependent covariates, such as average cinetic during the last 48 hours of the stay (slope) or Gaussian Processes kernels parameters, appear to have significant importances

**Fig. 5:**
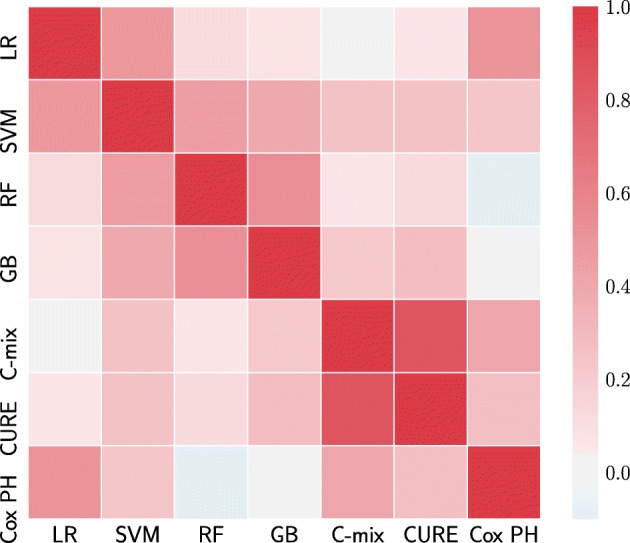
Pearson correlation matrix for comparing covariates selection similarities between methods. Red means high correlations

Let us now focus on the covariates selection aspect for each method. Figure 4 gives an insight on the covariates importance relatively to each model for 20 arbitrarily chosen covariates (selected on decreasing importance order for the C-mix model). The result with all covariates can be found in Section 3.3 of Additional file 1. One can observe some consistency between methods. Figure 5 gives a global similarity comparison measure in terms of covariates selection. We observe higher similarities between methods within a single setting.

## Discussion

In this paper, rather than trying to be exhaustive in terms of considered methods, we choose, accordingly with the aim of this paper, to offer a methodology for fairly comparing methods in the two considered settings. Also, we do not try different *ε* values, as it is done in [11] (where emphasis is on performance metrics), since our focus is to propose a general comparison and interpretation methodology, with an analysis that remains valid for any choice of *ε* value.

In the binary outcome setting, classifiers highly depend on how the risk groups are defined: a slight change of the survival threshold *ε* for assignment of classes can lead to different prediction results [11]. In our dataset, only 5.2% of the visits lead to a readmission within 30 days. We are then in a classical setup where the adverse event appears rarely in the data at our disposal. In such setting, a vast amount of temporal information is lost since the model only knows if a readmission occurs before the threshold delay or not. It appears that taking all the information through the survival analysis setting first, and then going back to a binary prediction using the survival estimate, significantly enhances any binary prediction, which intuitively makes sense.

Among all methods, the C-mix holds the best results. Its good performances compared to other methods is already shown in [10], both in synthetic and real data. While the Cox PH regression model is widely used to analyze time-to-event data, it relies on the proportional hazard ratio assumption. But in the case of VOC for instance, it is plausible that these early-readmissions are the consequences of the same ongoing crisis (hospital discharge before the VOC recovery), whereas late-readmissions are genuine new unrelated crisis (recurrence). This would suggest that the proportional hazard ratio assumption for Cox PH model (or its related models like the competing risks model, the marginal model or the frailty model; for this reason not considered in this study) is not respected in this situation. The CURE model main hypothesis being that a proportion of the patient is cured is questionable too. Those reasons partly explain the good performances of the C-mix model that does not rely on any restrictive hypothesis.

In this study, data extraction was performed with no a priori on the relevance of each variable. For instance, we extracted all biological covariates that have been measured during a patient’s stay, without presuming of their importance on readmission risk. Selected variables in our use case are relevant from a clinical point of view, highlighting the capacity of regularization methods to pinpoint clinically relevant covariates.

The most important covariates in the survival analysis setting are linked to the severity of the underlying SCD (e.g. crisis frequency, baseline hemoglobin), while selected covariates in the binary outcome setting are more related to the crisis biological parameters (e.g. arterial blood gas parameters). Some covariates appear to be selected in both settings (e.g. mean lactate deshydrogenase). All selected covariates make sens from a clinical point of view, and the difference between the two settings seems to be related to the underlying hypotheses of each setting: as binary setting only takes information on early readmission, crisis related parameters are favored; meanwhile in the survival analysis setting, parameters related to the severity of the underlying SCD are favored. This underlines why it is crucial, when working on prognosis analysis, to use several methods to get an insight of the most important covariates. Moreover, it insists on the fact that looking “only” at the diastolic blood pressure for instance – with an univariate point of view – would not be of any help to predict early readmission. Now, when considered within a high dimensional space (aka with a large number of other covariates) and using recent multivariate machine learning methods designed to extract and learn information from such complex high-dimensional setting, the same diastolic blood pressure could contribute to the prediction of patients at high risk of early readmission.

## Conclusions

In this paper, we compare methods in terms of prediction performances and covariates selection for different statistical and machine learning methods on a readmission framework with high dimensional EHR data. We particularly focus on comparing survival and binary outcome settings. Methods from both settings must be considered when working on a prognosis study. Indeed, important covariates are possibly different depending on the setting: for instance in our case study, we highlight important covariates linked either to the severity of the underlying SCD or to the severity of the crisis.

Not only do frequent readmissions affect SCD patients’ quality of life, they also impact hospitals’ organization and induce unnecessary costs. Our study lays the groundwork for the development of powerful methods which could help provide personalized care. Indeed, such early-readmission risk-predicting tools could help physicians decide whether or not a specific patient should be discharged of the hospital. Nevertheless, most selected covariates were derived from raw or unstructured extracted data, making it difficult to implement the proposed predictive models into routine clinical practice.

All results in the binary outcome setting rely on a critical readmission delay choice, which is a questionable - if not counterproductive - bias in readmission risk studies. Additionally, we point out the idea that learning in the survival analysis setting, rather than directly from the binary outcome setting, and then making binary predictions through the estimated survival function for a given delay threshold can dramatically enhance performances.

**Fig. 6:**
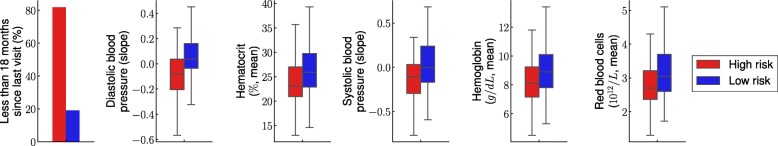
Covariates boxplot comparison between the most significant C-mix groups

Finally, the C-mix model yields the better performances and can be an interesting alternative to more classical methods found in the medical literature to deal with prognosis studies in a high dimensional framework. Moreover, it provides powerful interpretations aspects that could be useful in both clinical research and daily practice (see Fig. 6). It would be interesting to generalize our conclusions to external datasets, which is the purpose of further investigations.

## Additional file

Additional file 1 Supplementary material is given alongside the main manuscript, providing additional tables, figures or technical details mentionned in the manuscript. References to the right section of the Supplementary Material are precised throughout the paper and as soon as necessary. (PDF 444 kb)

## Abbreviations

AUC: Area under the (ROC) curve
CDW: Clinical data warehouse
CPOE: Computerized provider order entry
EHR: Electronic health record
GB: Gradient boosting
GPUH: George Pompidou university hospital
LOINC: Logical observation identifiers names and codes
NN: Artificial neural networks
PH: Proportional hazards
RF: Random forest
ROC: Receiver operating characteristic
SCD: Sickle-cell disease
SVM: Support vector machine
VOC: Vaso-occlusive crisis
