# Supplementary material for: Comparison of methods for early-readmission prediction in a high-dimensional heterogeneous covariates and time-to-event outcome framework
Source: BMC Med Res Methodol. 2019 Mar 6;19:50. doi: 10.1186/s12874-019-0673-4 (PMC6404305; doi:10.1186/s12874-019-0673-4)
Supplement: Supplementary file 1 — Supplementary material is given alongside the main manuscript, providing additional tables, figures or technical details mentionned in the manuscript. References to the right section of the Supplementary Material are precised throughout the paper and as soon as necessary. (PDF 444 kb) [file 12874_2019_673_MOESM1_ESM.pdf]

RESEARCH

Supplementary Material for the paper: Comparison of methods for early-readmission prediction in a high-dimensional heterogeneous covariates and time-to-event outcome framework

Simon Bussy[1*], Raphaël Veil[2,3], Vincent Looten[2,3], Anita Burgun[2,3], Stéphane Gaïffas[1,4], Agathe Guilloux[5], Brigitte Ranque[6,7] and Anne-Sophie Jannot[2,3]

*Correspondence:
simon.bussy@gmail.com
[1] Laboratoire de Probabilités Statistique et Modélisation (LPSM), UMR 8001, Sorbonne University, 4 Place Jussieu, 75005 Paris, France
Full list of author information is available at the end of the article

1 The case data

Let us motivate our choice of popuplation under study. We used this population because sickle cell disease is a worldwide health burden (the most frequent monogenic disorder), mostly in African population. Risk factors for readmission are not well understood, while for other chronic disease, many studies have been conducted on readmission. Moreover, focusing on a homogeneous population is more relevant in terms of clinical impact. Thus, there is a first clinical interest.

Moreover, we included a large number of covariates (high-dimensional setting) with no *a priori* hypothesis on which covariates should be important for predicting the readmission. Therefore, it was necessary to have a monocentric setting due to the heterogeneity of Electronic Heath Records (EHR) between different hospitals. This is particularly true for longitudinal variables, being a central focus in our study.

Indeed, very few studies in the literature are dedicated to the prediction of readmission in such a complex data space in terms of dimension or temporal dependency of the longitudinal covariates. And in this context, no one has yet compared recent machine learning methods simultaneously in the two theoretical settings used in readmission studies (survival analysis and binary classification) – while this is a paramount question – both in terms of prediction abilities and covariate selection for interpretation purposes.

Then, our sample is not very large in the context of chronic diseases, but all the retrospective studies about Vaso-Occlusive Crises (VOC) that use clinical data have a sample size of same order: see for instance Vichinsky et al. [7] with 538 patients (but this study is multicentric), Prasad et al. [6] with 58 patients, Frei-Jones et al. [4] with 100 patients, Darbari et al. [3] with 264 patients, Curtis et al. [2] with 432 patients (but this study focuses on emergency room only).

Finally, and most importantly, strictly in terms of methodology, all the methods used in this paper are actually designed for small sample size in a high-dimensional context. One key point of our paper is to propose a general methodology to compare and understand different models within distinct framework (survival or classification) for a given dataset, potentially leading to complementary conclusions and interpretations, and this message does not depend on the sample size of the dataset.

The pipeline proposed in our study is devoted to have a broader view than studying readmission rate for conditions with already well documented risk factors.

Because SCD is rare, patients are almost systematically addressed to hospital specialists for follow-up. In France, SCD experts are regrouped in SCD referral centers such as the GPUH. Therefore, in our case, primary care and urgent care are provided by the same hospital (the GPUH).

Hence, for patients included in this study, we had complete information regarding their follow-up and no alignment between primary care and hospital care was necessary. Moreover, our study focuses more on the methodological side than on readmission modeling for chronic diseases. The proposed pipeline holds for any hospitalized condition where readmission is of interest, not only chronic diseases.

2 Details on covariates

2.1 Descriptive data

Figure 1 represents the distribution of the number of stays.

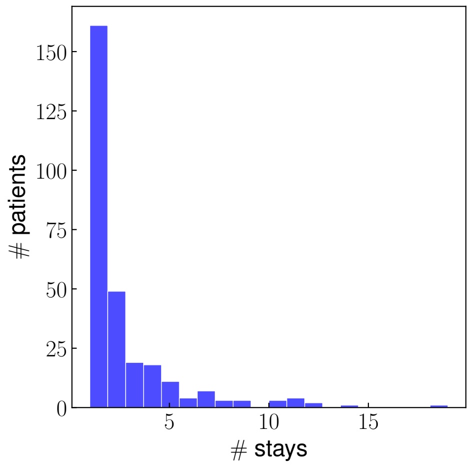

Figure 1: **Covariates selection similarities.** Number of stays per patient (# means “number of”). 161 patients have only one stay within the follow-up period, hence did not experience a readmission in this period (censored data).

The 161 patients with no readmission within the follow-up period are treated as censored ones in the survival analysis setting. This was possible because we had complete information on their follow-up.

2.2 Covariates creation

Since SCD patients are frequently treated with opioids to control the pain induced from VOCs, some may develop, over time, an addiction to these products. Such addiction may cause readmission and often interferes with hospitalization timeline. In order to limit confusion bias, we excluded patients encoded as opioid addicts (ICD-10 F11) as well as those who were treated with substitute products such as Methadone or Buprenorphine, both determined from hospitalization reports and drug prescriptions.

Regarding opioid treatment related information from the CDW, based on doctors and nurses inputs, variables extracted were the following:
- the specific molecule of each prescription,
- the specific dosage form of each prescription,
- the initiation and ending timestamps of each prescription.

From these variables, we also derived the following:
- the delay between the end of the last syringe received and the hospital discharge,
- the number of syringes used per day on average,
- the slope from the linear regression of the delay between syringes throughout the stay.

Regarding intravenous opioid treatments, we also extracted bolus dosage, maximum dosage, and refractory period. In order to capture both the average level and the general trend of these covariates, we derived them by calculating the slope and intercept from the linear regression of each of these parameters throughout the stay.

2.3 Missing data

We substitute missing medical history related data as follows: if a specific medical condition or VOC complication is mentioned in a report, this item is considered as part of the patient' medical history for every chronologically following stays; if a specific medical condition or VOC complication is explicitly stated as absent from the medical history in a report, this item is considered absent in all the previous stays.

For other specific covariates, we proceed that way:
- for the patients' baseline hemoglobin value, we use the last hemoglobin value measured during the first included stay,
- for the dichotomous variables regarding the patient's entourage and professional activity, we use the most represented value amongst all stays (of all patients),
- we consider non-mentioned medical history or VOC complications as absent,
- we consider that all patients received both opioid treatments and oxygen therapy at admission in the emergency room. Therefore, we consider the post-opioid observation period, as well as the post-oxygen observation period, to be the same time length as the entire stay.

For all remaining covariates, we impute as follows (after the random sampling of one stay per patient):
- numerical variables are imputed with their median values,
- categorical variables are imputed with their most represented values.

2.4 List of covariates

Table 2 summarizes the concepts used and their basic properties.

3 Details on experiments

3.1 Survival function estimation

For the Cox PH model, the survival $\mathbb{P}[T_i > t | X_i = x_i]$ for patient i in the test set is estimated by

$$\hat{S}_i(t|X_i = x_i) = [\hat{S}_0^{\text{cox}}(t)]^{\exp(x_i^\top \hat{\beta})},$$

where $\hat{S}_0^{\text{cox}}$ is the estimated survival function of baseline population ($x = 0$) obtained using the Breslow estimate of λ_0 [1]. For the CURE or the C-mix models, it is naturally estimated by

$$\hat{S}_i(t|X_i = x_i) = \pi_{\hat{\beta}}(x_i)\hat{S}_1(t) + \big(1 - \pi_{\hat{\beta}}(x_i)\big)\hat{S}_0(t),$$

where $\hat{S}_0$ and $\hat{S}_1$ are the Kaplan-Meier estimators [5] of the low and high risk of early-readmission subgroups respectively learned by the C-mix model (patients with $\pi_{\hat{\beta}}(x_i) > 0.5$ are clustered in the high risk subgroup, others in the low risk one), or cured and uncured subgroups respectively learned by the CURE model.

3.2 Hyper-parameters tuning

Let us summarize the hyper-parameters obtained after the cross-validation procedure for each method. First, we take $\eta = 0.1$ for all method using Elastic-Net regularization to ensure covariates selection. The strengh of the penalty is tuned to 42.81 for LR, 0.05 for SVM, 0.03 for C-mix, 0.008 for CURE and 0.014 for Cox PH. For RF, the maximum depth is 7, the minium sample's split is 3, the minimum sample's leaf is 2, the criterion is the entropy and the number of estimators is tuned to 200. For GB, the maximum depth is 7, the minimum sample's split is 3, the minimum sample's leaf is 4 and the number of estimators is 200. Finally for NN, the hidden layer's sizes is 3, the regularization term is tuned to 0.13.

3.3 Covariates importance comparison

Figure 2 gives the covariates importance estimates for all covariates and all considered methods.

4 Results in terms of accuracy and F-measure

Let us precise in Table 1 the results obtained in the binary outcome setting in terms of accuracy and F-measure, in addition to the AUC score.

Table 1: Comparison of prediction performances in the binary outcome setting for different metrics, with best results in bold.

Model	AUC	Accuracy (%)	F-measure
SVM	0.524	52.11	0.521
GB	0.561	54.59	0.547
LR	0.616	57.86	0.580
NN	0.707	70.24	0.701
RF	0.738	72.13	0.718
$\hat{S}^{\text{CURE}}$ ($\epsilon = 30$)	0.831	81.24	0.822
$\hat{S}^{\text{Cox}}$ ($\epsilon = 30$)	0.855	84.42	0.853
$\hat{S}^{\text{C-mix}}$ ($\epsilon = 30$)	**0.940**	**92.38**	**0.927**

Author details

[1] Laboratoire de Probabilités Statistique et Modélisation (LPSM), UMR 8001, Sorbonne University, 4 Place Jussieu, 75005 Paris, France. [2] Biomedical Informatics and Public Health Department, European Georges Pompidou Hospital, Assistance Publique-Hôpitaux de Paris, 20 Rue Leblanc, 75015 Paris, France. [3] INSERM, UMRS 1138 team 22, Centre de Recherche des Cordeliers, Université Paris Descartes, 15 Rue de l'École de Médecine, 75006 Paris, France. [4] CMAP, UMR 7641 École Polytechnique CNRS, Route de Saclay, 91128 Palaiseau, France. [5] LAMME, Univ Evry, CNRS, Université Paris-Saclay, France, 23 boulevard de France, 91025 Evry, France. [6] INSERM UMRS 970, Université Paris Descartes, 56 rue Leblanc, 75015 Paris, France. [7] Assistance Publique-Hôpitaux de Paris, Internal Medicine Department, Georges Pompidou European Hospital, 20 Rue Leblanc, 75015 Paris, France.

References

1. Norman E Breslow. Contribution to the discussion of the paper by dr cox. *Journal of the Royal Statistical Society, Series B*, 34(2):216–217, 1972.
2. Susanna A Curtis, Neeraja Danda, Zipora Etzion, Hillel W Cohen, and Henny H Billett. Elevated steady state wbc and platelet counts are associated with frequent emergency room use in adults with sickle cell anemia. *PLoS One*, 10(8):e0133116, 2015.
3. Deepika S Darbari, Zhengyuan Wang, Minjung Kwak, Mariana Hildesheim, James Nichols, Darlene Allen, Catherine Seamon, Marlene Peters-Lawrence, Anna Conrey, Mary K Hall, et al. Severe painful vaso-occlusive crises and mortality in a contemporary adult sickle cell anemia cohort study. *PLoS One*, 8(11):e79923, 2013.
4. Melissa J Frei-Jones, Joshua J Field, and Michael R DeBaun. Risk factors for hospital readmission within 30 days: a new quality measure for children with sickle cell disease. *Pediatric blood & cancer*, 52(4):481–485, 2009.
5. Edward L Kaplan and Paul Meier. Nonparametric estimation from incomplete observations. *Journal of the American statistical association*, 53(282):457–481, 1958.
6. Rajinder Prasad, Syed Hasan, Oswaldo Castro, Elliott Perlin, and Kyungsook Kim. Long-term outcomes in patients with sickle cell disease and frequent vaso-occlusive crises. *The American journal of the medical sciences*, 325(3):107–109, 2003.
7. Elliott P Vichinsky, Lynne D Neumayr, Ann N Earles, Roger Williams, Evelyne T Lennette, Deborah Dean, Bruce Nickerson, Eugene Orringer, Virgil McKie, Rita Bellevue, et al. Causes and outcomes of the acute chest syndrome in sickle cell disease. *New England Journal of Medicine*, 342(25):1855–1865, 2000.

Table 2: List of the considered concepts. For each one, we display the name (with unit), the category, the sub-category if relevant, and the type ("Q" for Qualitative, "B" for Binary and "C" for Categorical). For practical purposes, we only display basic concepts without describing the list of covariates induced from it and used in practice, since the process of covariates extraction is thoroughly described in the paper. For instance, the temperature concept gives rise to 5 covariates, relatively to its average and slope in the last 48 hours as well as the corresponding Gaussian Process kernel hyper-parameters.

Name (unit)	Category	Sub-category	Type	Name (unit)	Category	Type
Red blood cells ($10^{12}/L$)	Biological data	Complete blood count	Q	Respiratory rate (mvt/min)	Clinical data	Q
Hemoglobin (g/dL)	Biological data	Complete blood count	Q	Heart rate (bpm)	Clinical data	Q
Haemoglobin gap to baseline (g/dL)	Biological data	Complete blood count	Q	Oxygen saturation ($\%$)	Clinical data	Q
Hematocrit ($\%$)	Biological data	Complete blood count	Q	Temperature ($^\circ C$)	Clinical data	Q
Mean cell volume (fl)	Biological data	Complete blood count	Q	Post-oxygen observation period (hours)	Clinical data	Q
Mean corpuscular hemoglobin (pg)	Biological data	Complete blood count	Q	Systolic blood pressure ($mmHg$)	Clinical data	Q
Mean corpuscular hemoglobin concentration ($\%$)	Biological data	Complete blood count	Q	Diastolic blood pressure ($mmHg$)	Clinical data	Q
Reticulocytes ($10^9/L$)	Biological data	Complete blood count	Q	Gender	General features	B
Nucleated red blood cells ($10^9/L$)	Biological data	Complete blood count	Q	Baseline haemoglobin (g/dL)	General features	Q
White blood cells ($10^9/L$)	Biological data	Complete blood count	Q	Genotype	General features	B
Neutrophils ($10^9/L$)	Biological data	Complete blood count	Q	Distance between home and GPUH (km)	General features	Q
Neutrophils ($\%$)	Biological data	Complete blood count	Q	Driving time from home to GPUH (minutes)	General features	Q
Basophils ($10^9/L$)	Biological data	Complete blood count	Q	Age at hospital admission	General features	Q
Basophils ($\%$)	Biological data	Complete blood count	Q	French DRG code (GHM)	General features	C
Eosinophils ($10^9/L$)	Biological data	Complete blood count	Q	Severity level of the stay	General features	C
Eosinophils ($\%$)	Biological data	Complete blood count	Q	Length of hospital stay (hours)	General features	Q
Monocytes ($10^9/L$)	Biological data	Complete blood count	Q	Time length since last admission (days)	General features	Q
Monocytes ($\%$)	Biological data	Complete blood count	Q	Less than 18 months since last admission	General features	Q
Lymphocytes ($10^9/L$)	Biological data	Complete blood count	Q	Time length to next admission (days)	General features	Q
Lymphocytes ($\%$)	Biological data	Complete blood count	Q	Stayed in ICU	General features	B
Platelets ($10^9/L$)	Biological data	Complete blood count	Q	Number of RBC transfusions	General features	Q
Mean platelet volume (fl)	Biological data	Complete blood count	Q	Professional activity	Lifestyle	B
Hemoglobin S ($\%$)	Biological data	Hemoglobin electrophoresis	Q	Household situation	Lifestyle	B
Hemoglobin F ($\%$)	Biological data	Hemoglobin electrophoresis	Q	Acute chest syndrom	Medical history	B
Asparate transaminase (U/L)	Biological data	Liver function test	Q	Avascular bone necrosis	Medical history	B
Alanine transaminase (U/L)	Biological data	Liver function test	Q	Priapism (only for males)	Medical history	B
Alkaline phosphatase (U/L)	Biological data	Liver function test	Q	Ischemic stroke	Medical history	B
Gamma glutamyl-tranferase (U/L)	Biological data	Liver function test	Q	Leg skin ulceration	Medical history	B
Direct bilirubin (mol/L)	Biological data	Liver function test	Q	Heart failure	Medical history	B
Total bilirubin (mol/L)	Biological data	Liver function test	Q	Pulmonary hypertension	Medical history	B
Urea ($mmol/L$)	Biological data	Renal function test	Q	Known nephropathy	Medical history	B
Creatinine (mol/L)	Biological data	Renal function test	Q	Known retinopathy	Medical history	B
Renal function by MDRD ($mL/min/1,73m^2$)	Biological data	Renal function test	Q	Dialysis	Medical history	B
Sodium ($mmol/L$)	Biological data	Serum electrolytes	Q	Received Morphine	Opioid use	B
Potassium ($mmol/L$)	Biological data	Serum electrolytes	Q	Received Oxycodone	Opioid use	B
Chloride ($mmol/L$)	Biological data	Serum electrolytes	Q	Received orally administered opioids	Opioid use	B
Bicarbonate ($mmol/L$)	Biological data	Serum electrolytes	Q	Number of syringes received per day	Opioid use	Q
Total calcium ($mmol/L$)	Biological data	Serum electrolytes	Q	Delay between syringes (slope)	Opioid use	Q
Proteins (g/L)	Biological data	Serum electrolytes	Q	Post-opioid observation period (hours)	Opioid use	Q
Glucose ($mmol/L$)	Biological data	Serum electrolytes	Q	Bolus dosage	Opioid use	Q
C-reactive protein (mg/L)	Biological data	Other blood markers	Q	Maximum dosage	Opioid use	Q
Lactate Dehydrogenase (U/L)	Biological data	Other blood markers	Q	Refractory period	Opioid use	Q
Weight (kg)	Clinical data	Body dimensions	Q			
Size (cm)	Clinical data	Body dimensions	Q			
Body mass index (kg/m^2)	Clinical data	Body dimensions	Q			

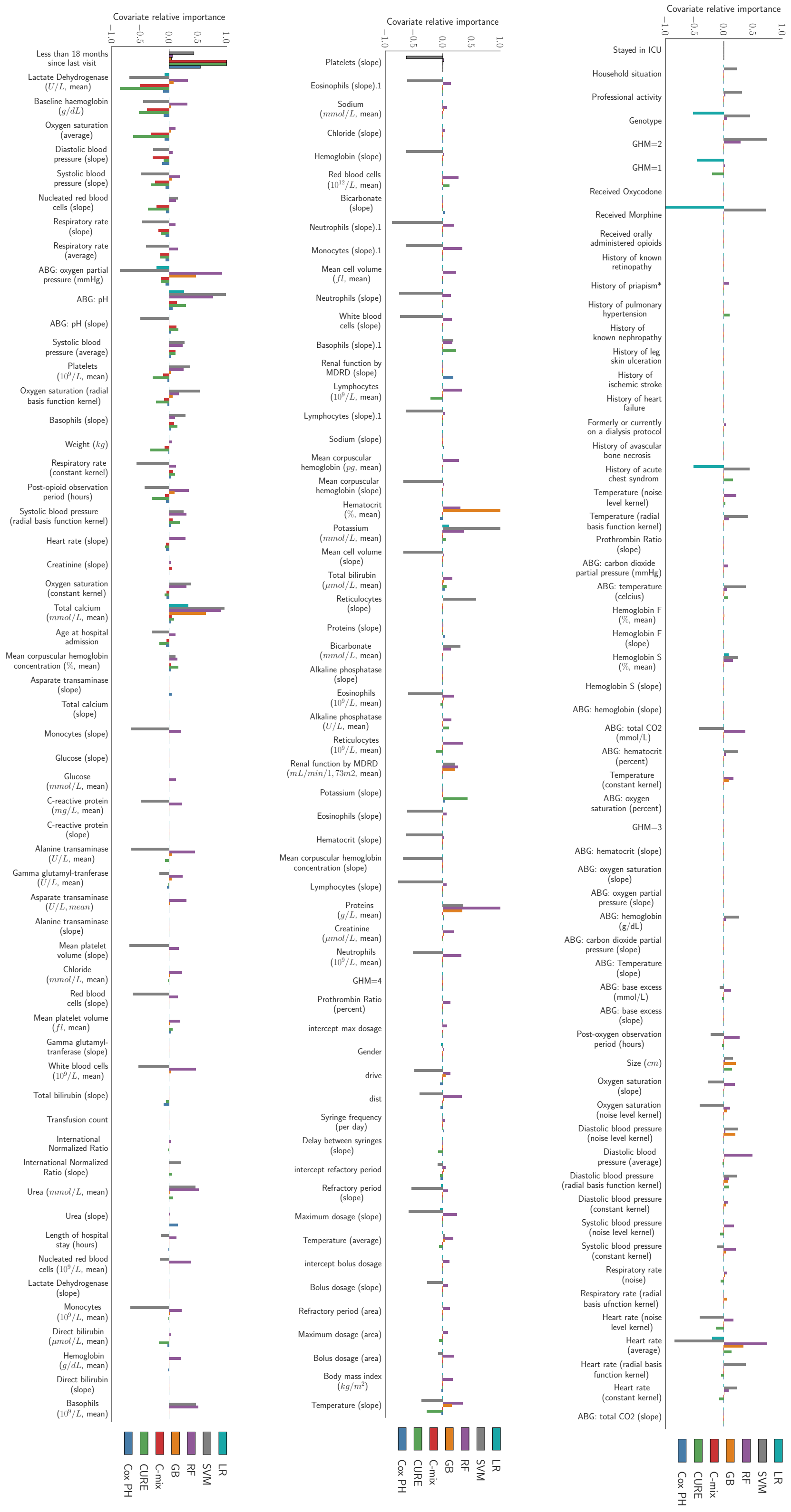

Figure 2: **Covariates importance.** Comparison of covariates importance, ordered on the C-mix estimates. Note that for RF and GB models, coefficients are, by construction, always positive.
